# Biological sex as a variable in immunity does not affect parabolic flight-induced alterations in immune responses

**DOI:** 10.3389/fimmu.2025.1673072

**Published:** 2025-11-17

**Authors:** Dominique Moser, Judith-Irina Buchheim, Katharina Biere, Sandra Matzel, Federico D´Amico, Alexander Choukér, Tobias Woehrle, Matthias Feuerecker

**Affiliations:** Laboratory of Translational Research ‘Stress and Immunity’, Department of Anesthesiology, LMU Hospital, Ludwig-Maximilians-University Munich, Munich, Germany

**Keywords:** gravitational stress, parabolic flight, sex-specific medicine, whole-blood incubation assay, innate and adaptive immunity

## Abstract

The present era of spaceflight is accompanied by two meaningful breakthroughs. The access to in-orbit missions for civilians and the increasing enrolment of female astronauts require detailed investigations on the impact of gravitational stress on human physiology with focus on sex-specific differences. To assess the responsive capacities of innate and adaptive immunity in this context, functional characterizations were performed in women and men in a parabolic flight study. Blood and saliva were collected 1 month prior and on the day before the flight, as well as directly after flight and on the following day. Leukocyte proportions were quantified, and NETosis as well as phagocytic activity was tested. The impact of gravitational stress on the ability to mount a functional immune response was examined by a 6-h whole-blood incubation assay with subsequent analysis of leukocyte surface activation marker expression and cytokine secretion. Parabolic flight induced a temporary increase in granulocyte proportions, which however did not influence NETosis and phagocytosis. Throughout the flight week, leukocyte activation and cytokine secretion patterns remained unaltered in response to antigen stimulation. No differences were found regarding the direction or intensity of immune response either in women or in men. However, when comparing effects 1 month before flight and the flight week, immune responses were still present but remarkably weaker during flight week, which was independent of cortisol levels. Altogether, this study elicited two important findings. Firstly, no sex-specific increased risk exists for immune dysregulation by acute gravitational stress. Secondly, merely changing the day to day surrounding dampens crucial immune responses, which requires further investigations.

## 1 Introduction

Technical advances in the last decade enabled conducting short-term missions aboard the International Space Station (ISS) such as the Axiom Missions, during which private astronauts spent up to 14 days in Low Earth Orbit (LEO) to perform science, outreach, and commercial activities (1, 2). Moreover, these advances shifted the privilege of participating in spaceflight missions from well-selected and trained astronauts to civilians. Especially in the 2020s, several commercial spaceflight missions were launched such as Inspiration4, Polaris Dawn (3, 4), and Fram2 (5), lasting between 3 and 5 days with a crew size of four humans, including both male and female participants.

In light of these short-term missions as well as of long-term missions aboard the ISS (6 months in average) or future deep-space exploration missions, the impact of gravitational alterations on human physiology remains the subject of intricate investigations. Both microgravity (µ*g*) and hypergravity impact neurovestibular and cardiovascular systems, induce fluid shifts, and affect the musculoskeletal apparatus (6–10). Moreover, the condition of microgravity in particular has been shown to induce dysfunctions of the immune system, leading to increased susceptibility towards infection and reactivation of dormant viruses, or paradoxically to hypersensitivity reactions (11–13).

Experiments to study the effects of very acute physiological alterations induced by gravitational stress are not feasible during spaceflight or directly after return to Earth due to logistical constraints. Parabolic flights (PF) offer the possibility to study these effects in an Earth-bound short-term model. Within such flight manoeuvres, the aircraft is directed in a parabolic trajectory, leading to 22 s of microgravity that is flanked by 20 s of 1.8*g* hypergravity, each in a sequence of 31 parabolas (14–16). Arising gravitational stress was observed to induce indeed physiological alterations as recognized in space, at least to a certain extent (15). At the immunological level, for instance *ex vivo* investigations revealed a sensitization of innate immunity, which was demonstrated by a primed state of polymorphonuclear granulocytes (17) and monocytes (18).

Because of the historical consideration of men being the “normal case” in medicine, and because of the numerical dominance of male astronauts, most studies on human physiology under spaceflight conditions enrolled mainly male study participants.

However, there is a visibly growing enrolment of female astronauts as represented aboard the ISS. Additionally, equal sex distribution is planned for future deep-space missions as well as already represented by the gender balanced crew compositions during commercial space flights. This development demonstrates the necessity of characterizing sex-specific reactions to gravitational stress thoroughly in order to apply tailored countermeasure approaches in the case of disease, according to the principles of sex-specific medicine. Regarding immunity, women generally show more intense immune responses, which is represented by a higher pathogen clearance and vaccine efficiency but also by an increased susceptibility of developing autoimmune reactions, which is in particular due to higher CD4^+^ T-cell and B-cell counts (19–22), suggesting the hypothesis of a differential impact of acute gravitational stress on immune responses in women and men.

To address this, the present study was conducted to evaluate sex-specific differences in responsive capabilities of the innate and adaptive immunity after PF.

## 2 Materials and methods

### 2.1 Study population and protocol

After giving their informed consent, 23 healthy volunteers (14 women, 9 men) participated in two PF campaigns provided by the German Space Agency (DLR) and executed by the French company Novespace. The study subjects’ demographic data are summarized in **Table 1**. These campaigns took place in June 2021 and February 2022. Due to the COVID-19 pandemic, both campaigns were conducted starting from the airport Paderborn-Lippstadt, Germany, and not as usual from Bordeaux, France. This led to an additional flight time of approximately 1 h. The initial study protocol foresaw no medication with scopolamine to prevent motion sickness for study participants. Due to the increased flight time and unavailability of scopolamine medication on the plane, the responsible study team coordinators decided to give every participant the opportunity to receive scopolamine upon demand via a subcutaneous injection. All subjects made use of the scopolamine injection. Additionally, all passengers had to wear FFP2 facial masks during the duration of the flight and adhere to standard preventive measures (e.g., hand disinfection, daily measurement of body temperature, negative COVID-19 qPCR 24h upon arrival, daily COVID-19 antigen testing, limited social contact).

**Table 1:** Demographic data of female (n=14) and male (n=9) study participants.

Characteristic	All	Women	Men
Age (y)	34.7 ± 4.5 (29-47)	33.3 ± 2.7 (29.6-40.3)	36.9 ± 5.4 (29-47)
Size (m)	1.72 ± 0.08 (1.58-1.85)	1.68 ± 0.06 (1.58-1.82)	1.80 ± 0.05 (1.64-1.85)
Weight (kg)	71.2 ± 13.5 (55-108)	63.7 ± 6.3 (55-77.5)	83.01 ± 13.3 (64-108)
BMI (kg/m^2^)	23.9 ± 3.1 (19.5-31.9)	22.7 ± 1.8 (19.5-25.1)	25.7 ± 3.7 (21.3-31.9)
Sex (f/m)	14/9	14	9

Values are given as mean ± SD as well as data range. BMI, Body mass index.

The PF was performed with an Airbus A310 plane. Per PF campaign, flights were performed on 3 days. On each flight day, 1 test parabola and 30 regular parabolas were completed. One parabola consists of a first hypergravity period (approx. 20-s pull-up), followed by a 22-s lasting microgravity phase, and ended by another hypergravity sequence (1.8*g*) (see also www.airzerog.com). Due to a technical issue during the here reported first PF campaign, one flight day had to be postponed.

The study protocol consisted of sample collections 1 month before the scheduled campaign (baseline data collection (BDC), in the morning), at the day before the flight (L-1, in the morning), at the morning of the flight (T0), and at four time points during the flight (before the first parabola (T1); after parabola 10/15 (T2); after parabola 20/25 (T3); after completion of all parabolas (T4)), after landing (T5), in the evening of the flight day (T6), and at the day after the flight (R+1, in the morning). For the presented investigations venous blood was drawn from the forearm and saliva was collected in salivettes (51.1534.500, Sarstedt, Nümbrecht, Germany) at time points BDC, L-1, T5, and R+1. For all sample collections performed in the morning, participants were not allowed to have food 6 h prior or to drink clear fluids 2 h before.

This investigation was conducted in accordance with the ethical norms and standards of the Declaration of Helsinki and its revisions (23). The LMU Munich Medical Faculty ethics committee, Germany, approved this study (Protocol Nr. 19-846). In addition, the Ethics committee of the University of Caens, France, gave approval for the realization of the here presented investigation.

The study team installed a fully equipped laboratory inside the airport, including centrifuges, fridge/freezer combination (+6/−20°C), incubators, and on-site flow cytometry (Guava^®^ easyCyte, Merck). Samples were processed immediately after collection and hence allowed live cell analyses. To obtain the best possible results, aliquoted samples were stowed on dry ice at a temperature of ~−70°C and transported to the investigator’s laboratory for final storage at −80°C.

### 2.2 Blood parameters

#### 2.2.1 Complete blood count

Leukocyte subpopulations were measured by complete blood count (CBC) at the Institute of Laboratory Medicine at the University of Munich, Germany, within 24h after blood draw.

#### 2.2.2 NETosis

Primary neutrophils were isolated from whole blood using EasySep™ Direct Human Neutrophil Isolation Kit (StemCell Technologies, Vancouver, Canada) by immunomagnetic negative selection, which keeps desired neutrophils untouched whereas unwanted cells are targeted and removed by antibody complexes and magnetic particles. Cell number and viability were quantified by TC20 automated cell counter (Bio-Rad, Hercules, CA, USA). NETosis was assessed by Cayman’s NETosis Assay Kit (Cayman Chemicals, Ann Arbor, MI, USA) by measuring neutrophil elastase activity according to the manufacturer’s guide. Of the negatively isolated primary neutrophils, 1×10^6^ viable cells were seeded per well (12-well plate) and stimulated for 3 h with phorbol myristate acetate (PMA) (20 nM), ATP (0.5 mM), ATP+PMA, lipopolysaccharide (LPS) (10 pg/ml), or LPS+ATP to release neutrophil extracellular traps (NETs).

#### 2.2.3 Phagocytosis assay

Phagocytic activity in whole blood was detected by pHrodo™ Green BioParticles™ Phagocytosis Kit by flow cytometry using zymosan and *E. coli* bioparticles (Thermo Fisher, Waltham, MA, USA). The experiment protocol was conducted according to the manufacturer’s instructions.

#### 2.2.4 BD IMK Multitest kit

To determine the different lymphocyte subsets, the commercially available BD Multitest™ IMK kit (Cat. No. 340182, BD Biosciences, Sn Jose, CA, USA) for flow cytometric analyses was performed in accordance with the manufacturer’s guidelines. This kit allows to measure the percentages and absolute counts of mature human lymphocyte subsets (T lymphocytes (CD3^+^), B lymphocytes (CD19^+^), helper/inducer T lymphocytes (CD3^+^CD4^+^), suppressor/cytotoxic T lymphocytes (CD3^+^CD8^+^), and natural killer (NK) lymphocytes (CD3^–^CD16^+^and/or CD56^+^).

#### 2.2.5 *Ex vivo* whole-blood incubation assay

Lithium-heparin anticoagulated whole blood was diluted with an equal volume of RPMI 1640 (Sigma-Aldrich, Steinheim, Germany) cell culture medium and stimulated with one of the following stimuli: lipopolysaccharide (LPS; 10 ng/ml; Sigma-Aldrich, Steinheim, Germany), heat-killed *Listeria monocytogenes* (HKLM; 10^8^ cells/ml; InvivoGen Europe, Toulouse, France), or CD3/CD28 activator (25 µl/ml; STEMCELL Technologies, Vancouver, BC, Canada). For negative control, stimulation was performed with cell culture medium only (basal). Samples were incubated for 6 h at 37°C. Following incubation, supernatants were collected and stored at −80°C until cytokine measurement. The remaining sample was conserved with Transfix (Cytomark, Buckingham, UK) and stored at 4°C until flow cytometry analyses.

#### 2.2.6 Immunophenotyping

For monocyte surface activation marker analysis, leukocytes were stained for co-expression of CD14 and CD69, HLA-DR, TLR2, TLR4, CD40, CD80, or CD86. For neutrophilic granulocytes, CD16^+^ cells were stained for co-expression of CD62L. For T cells, CD4^+^ or CD8^+^ cells were stained for co-expression of CD69 and CD28. Except for TLR2-APC (Cat. No. 130-120-052, Miltenyi Biotec, Bergisch Gladbach, Germany), TLR4-PerCp (FAB6248C, R&D Systems, Minneapolis, MN, USA), and CD80-APC (A15707, Invitrogen, Waltham, MA, USA), all antibodies were obtained from BD Biosciences (Cat. No. CD4-FITC: 555346, CD8-APC: 555369, CD69-PE: 555531, HLA-DR-APC: 559866, CD28-PerCp-Cy5.5: 337181, CD14-FITC: 345784, CD16-PerCp: 338440, CD69-PerCp: 340548, CD40-PE: 555589, CD86-PE: 555665, CD62L-PE: 555544, Franklin Lakes, NJ, USA). Samples and antibodies were incubated for 20min at room temperature, lysed for 10min (BD FACS lysing solution, BD Biosciences Franklin Lakes, NJ, USA), washed with PBS, and analysed by flow cytometry (Guava^®^ easyCyte™ 8HT Flow Cytometer, Merck Millipore, Billerica, MA, USA). For each measurement, 10,000 events were recorded. Data analysis was performed with InCyte Software for Guava^®^ easyCyte HT Systems (Merck Millipore, Billerica, MA, USA).

#### 2.2.7 Cytokine measurements

Cytokine concentrations were quantified from thawed *ex vivo* incubation assay supernatants using the MAGPIX Multiplexing System (Luminex, Austin, TX, USA) and custom-made Multiplex assays (Merck Millipore, Billerica, MA, USA) for detection of G-CSF, GROα, IL-1β, IL-1RA, IL-2, IL-6, IL-8, IL-10, TNF, and IFNγ according to the manufacturer’s instructions.

#### 2.2.8 Cortisol ELISA

Salivary cortisol concentrations were measured using the Human Cortisol Competitive ELISA Kit (EIAHCOR, Thermo Fisher, Waltham, MA, USA) according to the manufacturer’s protocol.

#### 2.2.9 Statistical analyses

The data were tested for normal distribution using the Shapiro–Wilk test. To realize within-group comparisons over time (e.g., changes in cytokine concentrations over time in one sex), one-way repeated measure analysis of variances (one-way RM-ANOVA) was applied. Within-time-point comparisons for different stimuli per group (e.g., whole-blood incubation assay) were calculated by one-way ANOVA. Tests were followed by the *post-hoc* Holm–Sidak, Tukey, or Dunn’s test to correct for multiple comparisons. Between-group comparisons (between the two sexes) were executed using a t-test for normally distributed data and the Mann–Whitney *U* test for non-parametric data. The mean differences were considered significantly different if p < 0.05 and are indicated as such on each data table and figure. Statistical calculations were performed using SigmaPlot^®^ 12.5 (Systat Software, Chicago, IL, USA).

## 3 Results

### 3.1 White blood cell count and proportions of leukocyte subsets

**Figure 1:**
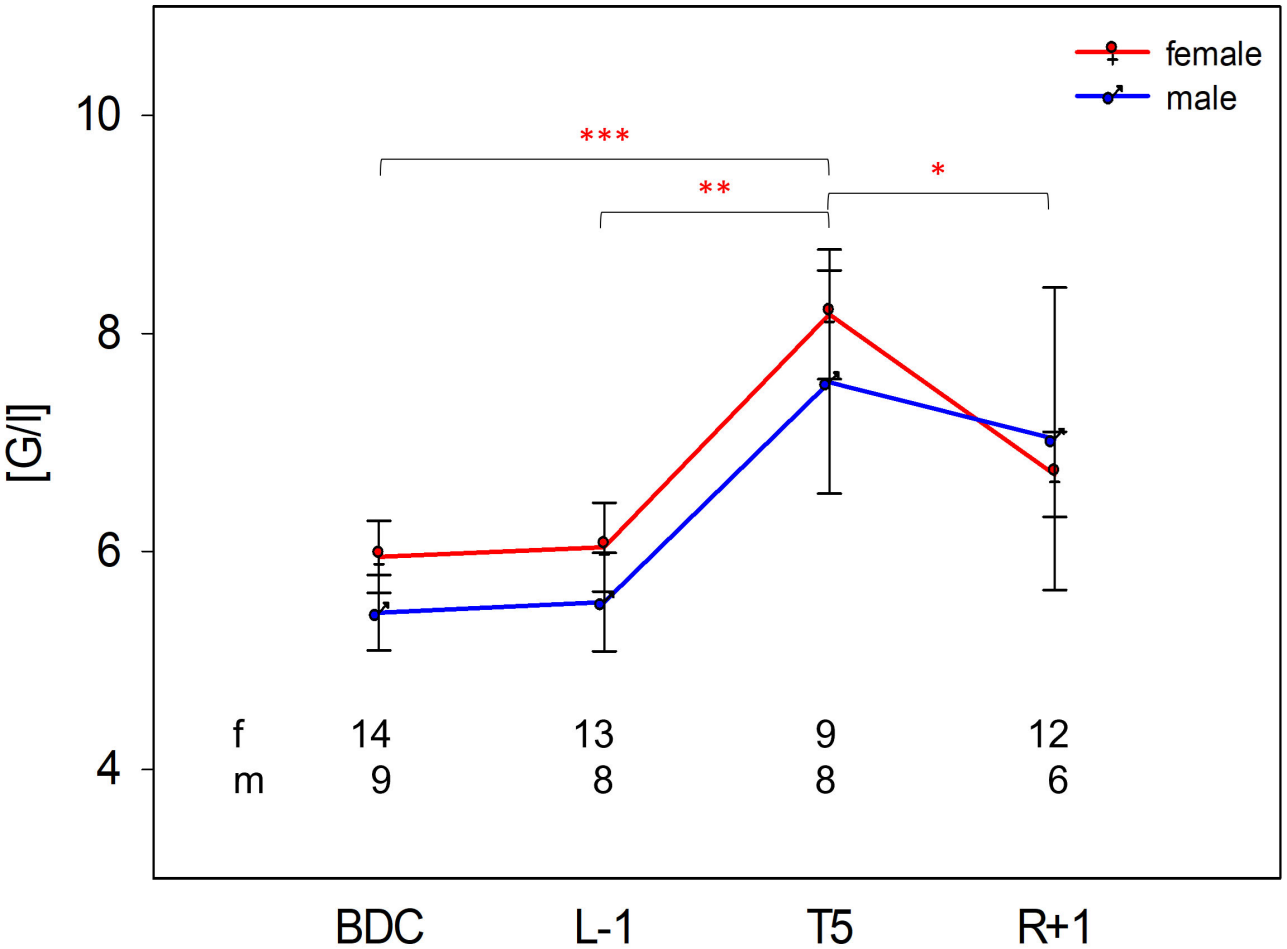
Absolute white blood cell counts [G/l] in female and male study participants at time points BDC, L-1, T5, and R+1. Numbers within the plot indicate analysed study subjects per time point. Differences between study time points within the same group were calculated by one-way repeated measures ANOVA followed by *post-hoc* Holm–Sidak test. Significant differences occurred only in the female group and are indicated in red asterisks. *P <.05, **P <.01, ***P <.001.

In order to comprehend immune functional alterations caused by acute gravitational stress in dependence of biological sex, blood cell count, as well as leukocyte subset analysis, was performed at two pre- (BDC, L-1) and two post-flight (T5, R+1) time points. PF induced in both groups an increase in white blood cell numbers, reaching statistical significance in women at T5 compared to BDC, L-1 and R+1, but not in men. One day after PF, values remained at higher levels than pre-flight in both groups (**Figure 1**).

**Figure 2:**
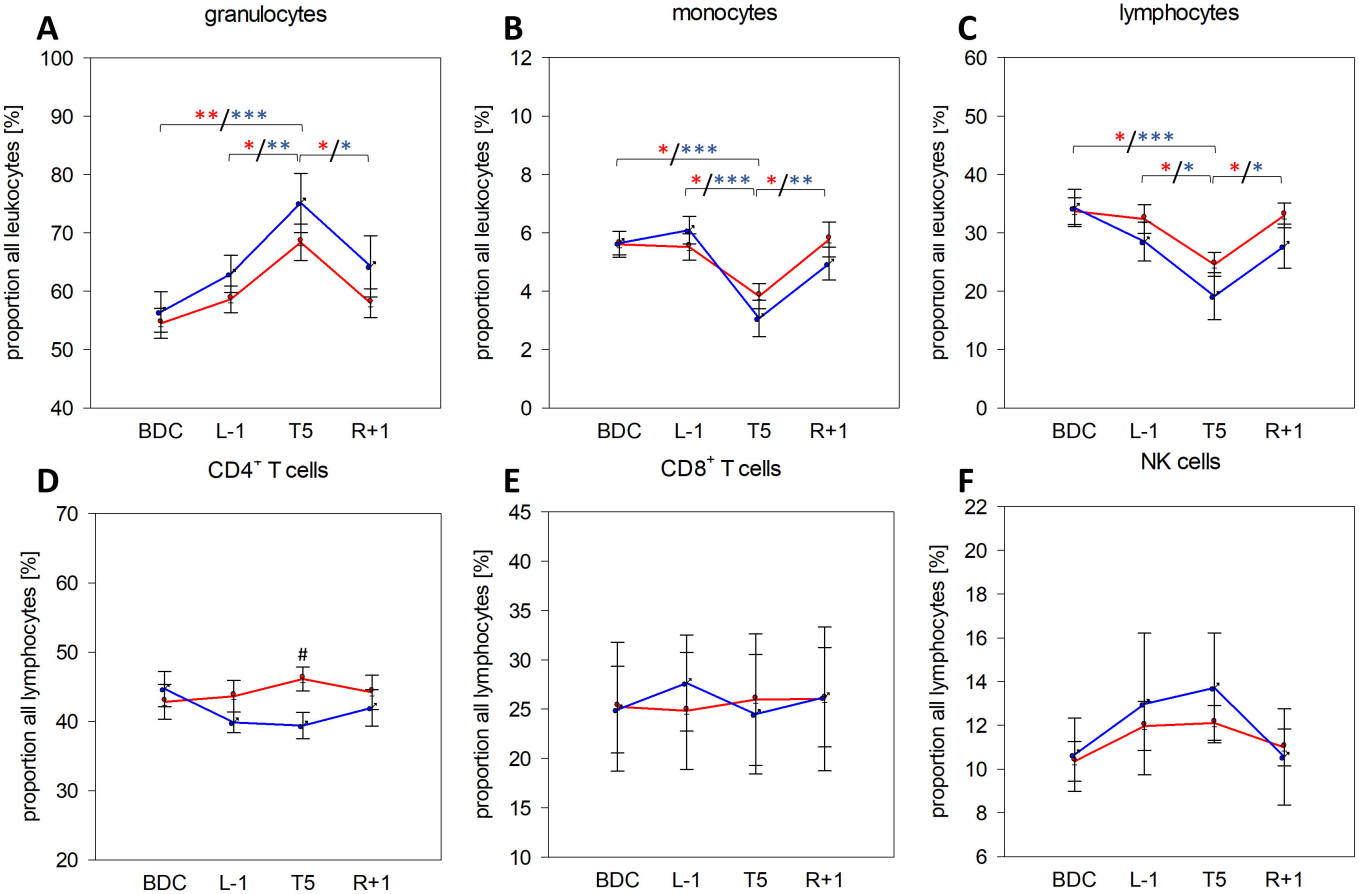
Proportions of granulocytes **(A)**, monocytes **(B)**, and lymphocytes **(C)** among all leukocytes in whole blood as well as of CD4^+^ T cells **(D)**, CD8^+^ T cells **(E)**, and NK cells **(F)** among all lymphocytes at the respective sample collection time points using IMK Multitest. Plots represent mean values ± SD in women (red, n=14 (BDC, L-1, T5), n=13 (R+1)) and men (blue, n=9 (BDC, T5), n=8 (L-1), n=6 (R+1)). Differences between groups were calculated using unpaired two-tailed Student’s *t* test (#). Differences between study time points within the same group were calculated by one-way repeated measures ANOVA (*) followed by *post-hoc* Holm–Sidak test, whereby differences in the female group are indicated in red and in the male group in blue asterisks. ^#^/*P <.05, **P <.01, ***P <.001.

Leukocyte subset analysis showed a clear and significant increase in granulocyte proportions at T5 in both groups (**Figure 2A**), whereas monocyte and lymphocyte proportions were significantly lower at T5 compared to all other time points (**Figures 2B, C**). For these three subsets, effects were more pronounced in men, however without significant differences between groups. B-cell proportions were slightly elevated in men but barely affected by PF (**Supplementary Table 1**). CD4^+^ T-cell proportions showed an increasing trend in women within the analysis time points whereas proportions were declining in men, resulting in significantly higher CD4^+^ T-cell proportions in women at T5 (**Figure 2D**). CD8^+^ T-cell proportions were unaffected by PF and without measurable sex-specific differences (**Figure 2E**). NK cell proportions tended to be higher on the flight day without showing significance between time points or sexes (**Figure 2F**). The observed increase in granulocyte proportions and decrease in monocyte and lymphocyte proportions complied with measured relative CBC values (**Table 2**) and were reflected by increased neutrophil-to-lymphocyte ratios (NLR), which were significantly increased in women at T5 (mean ± SD: 2.60 ± 0.91) compared to all other time points (mean values: BDC: 1.64 ± 0.60 (*P*= .011), L-1: 1.64 ± 0.36 (*P*= .012), R+1: 1.84 ± 0.85 (*P*= .047) and at T5 (4.82 ± 3.46) compared to BDC (1.83 ± 0.66) in men (*P*= .034).

**Table 2:** Relative values of neutrophils, monocytes, and lymphocytes per sample collection time point determined by complete blood cell count.

Time point	Neutrophils [%]		Monocytes [%]		Lymphocytes [%]	
	♀	♂	♀	♂	♀	♂
BDC	55.0 ± 12.9	55.2 ± 8.8**	7.6 ± 1.9	8.7 ± 2.5*	35.4 ± 9.9**	32.8 ± 7.7
L-1	53.8 ± 4.9*	61.8 ± 15.7	7.1 ± 3.2	6.8 ± 2.8	36.5 ± 7.9**	31.0 ± 14.2
T5	64.3 ± 9.3	72.1 ± 12.9	6.3 ± 2.3	5.9 ± 2.3	24.1 ± 8.9	21.0 ± 11.0
R+1	55.0 ± 10.3	61.6 ± 8.4	6.5 ± 2.7	7.2 ± 3.6	32.8 ± 8.7**	25.2 ± 8.2

Values are given as mean ± SD. N-numbers at BDC, L-1, T5, R+1: neutrophils: 14/9/7/8 (women) and 9/5/7/5 (men); monocytes: 14/13/8/12 (women) and 9/8/7/5 (men), lymphocytes: 14/13/9/12 (women) and 9/8/7/6 (men). Differences of T5 to other study timepoints within the same group were calculated by one-way repeated measures ANOVA followed by *post-hoc* Holm–Sidak test. *P <.05, **P <.01.

### 3.2 Analysis of innate immune cell functions

Based on the observation of increased granulocyte proportions with a concomitant proportional reduction of monocytes after PF, the basic innate immune cell functions of NETosis and phagocytosis were examined.

**Figure 3:**
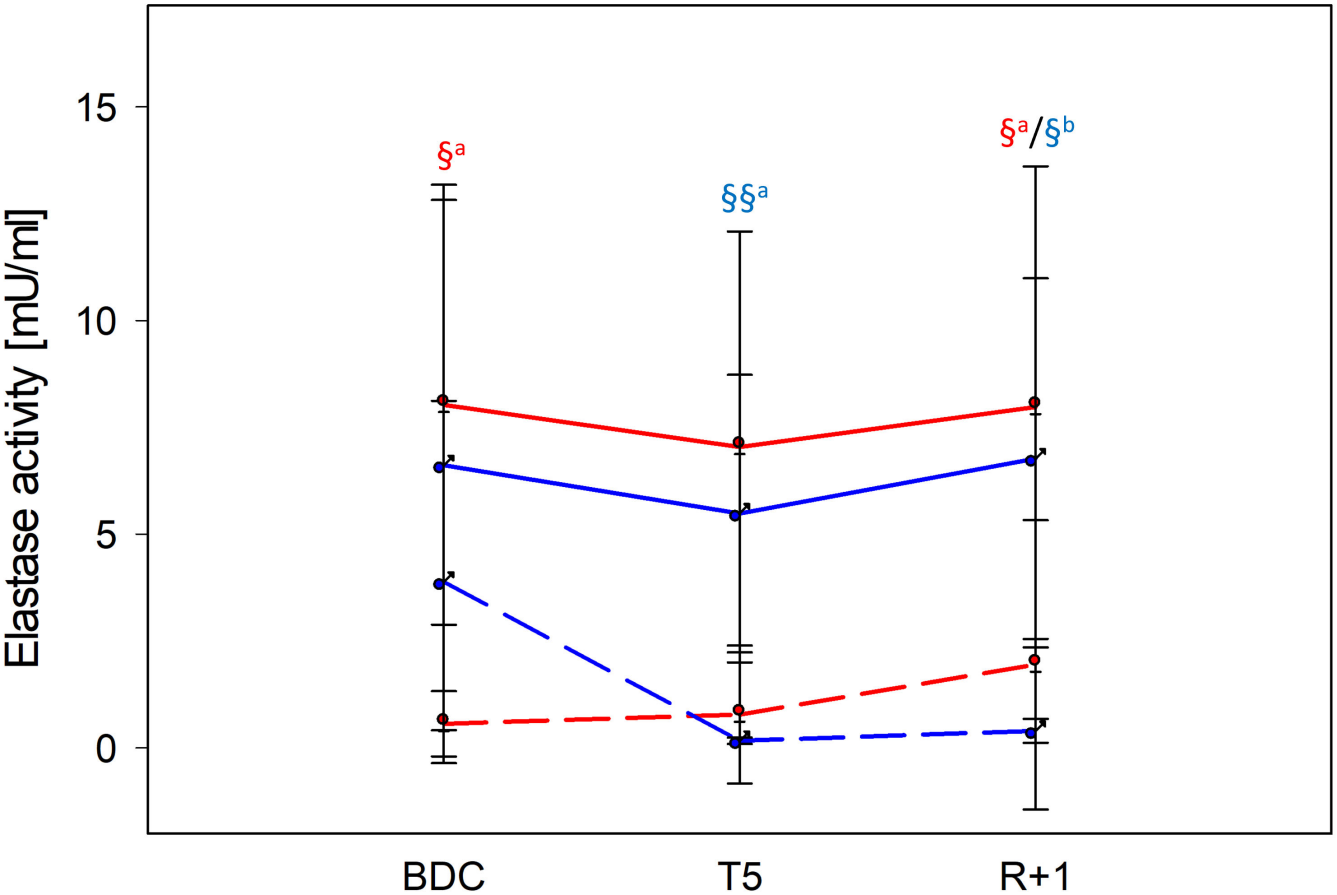
NETosis intensity at time points BDC, T5 and R+1 quantified by neutrophil elastase activity [mU/ml]. Neutrophils were incubated for 3 h with phorbol 12-myristate 13-acetate (PMA) for NETosis induction. Values show mean ± SD for women (red lines, n=9) and men (blue lines, n=5). Differences between negative control (dashed lines) and PMA (filled lines) within the same group were calculated by one-way ANOVA followed by *post-hoc* Tukey test (^a^) or Holm–Sidak (^b^) test. ^§^P < 0.05, ^§§^P < 0.01.

For NETosis induction, negatively separated neutrophils were incubated with different potent stimuli, and neutrophil elastase activity, a hallmark of NETosis, was quantified. From the applied stimuli, only incubation with PMA induced well-measurable NETosis (**Figure 3**, **Supplementary Figure 1**). Elastase activity by PMA was similar in men and women, reaching significant differences to untreated control values at BDC and R+1 in women, whereas in men, significance was achieved at T5 and R+1. In both groups, NETosis capacities were unaffected by PF (**Figure 3**).

Phagocytic activity was examined by the uptake of fluorochrome-labelled zymosan and *E. coli* particles. Both particle types were taken up by leukocytes resulting in significant fluorescent signals, with phagocytosis of *E. coli* particles being more pronounced than that of zymosan particles. Significant differences were neither observed between time points nor between sexes (**Table 3**).

**Table 3:** Phagocytic activity in whole-blood samples after incubation with fluorochrome-labelled zymosan and *E. coli* particles.

Time point	Negative control		Zymosan		E. coli	
	♀	♂	♀	♂	♀	♂
BDC	31.70 ± 9.36	33.84 ± 8.67	94.04 ± 23.56^§§^	112.47 ± 47.32^§^	261.49 ± 151.43^§§§^	333.13 ± 176.46^§§§^
L-1	27.70 ± 8.59	27.81 ± 9.87	112.44 ± 73.82^§§^	113.96 ± 42.92^§^	196.79 ± 43.88^§§§^	303.62 ± 180.86^§§§^
T5	24.36 ± 9.4	24.01 ± 8.75	118.81 ± 49.81^§§^	103.71 ± 48.69^§^	223.70 ± 73.15^§§§^	221.76 ± 73.60^§§§^
R+1	24.48 ± 9.73	25.06 ± 14.76	85.90 ± 54.41^§§^	76.53 ± 30.15	204.77 ± 70.10^§§§^	200.75 ± 86.34^§§§^

Values represent MFIs (mean fluorescent intensities) and are shown as mean ± SD. Analyses were performed at BDC, L-1, T5, and R+1. Women: n = 14 (13 on R+1); men: n = 9 (6 on R+1). Differences between negative control and stimulus within the same group were calculated by one-way ANOVA followed by *post–hoc* Tukey test. ^§^P < 0.05, ^§§^P < 0.01, ^§§§^P < 0.001.

### 3.3 Analysis of leukocytes’ activation state

Changes in spontaneous immune cell activation by PF were assessed by quantification of surface activation marker expression on monocytes (CD14^+^), granulocytes (CD16^+^), and T cells (CD4^+^ and CD8^+^).

Analysis of surface markers on monocytes displayed significantly higher CD40 expression at L-1 and R+1 than at BDC and T5 in female study participants. In addition, HLA-DR expression was lower at L-1 than measured at BDC in women as well as HLA-DR and TLR2 expression at R+1. Expression of CD62L on granulocytes was unaffected by PF. T-cell subsets displayed subliminal but still significant fluctuations of CD69 and CD28 expression in both groups (**Table 4**).

**Table 4:** Native cell surface expression of cell activation markers on CD14^+^ monocytes, CD16^+^ granulocytes, and CD4^+^ and CD8^+^ T cells.

Marker		BDC		L-1		T5		R+1	
		♀	♂	♀	♂	♀	♂	♀	♂
CD14	CD40	6.13 ± 3.98	8.05 ± 2.55	14.47 ± 4.86^***,§§§^	12.14 ± 6.03	6.90 ± 2.64	7.60 ± 3.88	15.55 ± 6.77^***,§§§^	14.68 ± 8.33
	CD69	3.59 ± 1.86	4.11 ± 2.99	5.71 ± 2.98	5.90 ± 6.69	3.56 ± 2.33	2.38 ± 1.72	5.77 ± 2.98	4.64 ± 4.98
	CD80	3.55 ± 2.82	4.52 ± 3.42	3.76 ± 3.38	4.45 ± 3.18	2.42 ± 1.49	1.97 ± 1.29	3.32 ± 1.13	3.05 ± 3.83
	CD86	68.32 ± 9.58	59.55 ± 17.72	55.66 ± 13.69	49.52 ± 12.85	68.18 ± 16.21	62.95 ± 13.41	66.44 ± 17.71	66.37 ± 9.59
	TLR2	93.53 ± 4.73	93.66 ± 3.48	88.48 ± 6.14	89.55 ± 5.33	91.04 ± 4.83	92.76 ± 2.39	86.85 ± 6.64^*^	88.71 ± 5.57
	TLR4	10.88 ± 3.64	11.90 ± 3.33	14.03 ± 4.97	13.90 ± 6.78	11.52 ± 3.92	13.88 ± 9.14	12.94 ± 3.62	11.45 ± 3.11
	HLA-DR	79.83 ± 9.80	81.66 ± 5.65	61.08 ± 14.07^***^	66.88 ± 12.76	68.30 ± 14.73	69.29 ± 18.51	66.98 ± 12.19^*^	66.46 ± 12.36
CD16	CD62L	48.25 ± 18.18	36.72 ± 17.39	43.92 ± 16.90	45.16 ± 20.18	43.23 ± 17.60	47.37 ± 20.19	44.80 ± 21.42	49.42 ± 29.87
CD4	CD69	1.97 ± 1.24	2.04 ± 0.80	3.34 ± 2.11	1.80 ± 0.92^#^	2.70 ± 1.87	1.93 ± 1.24	3.43 ± 3.23	1.72 ± 0.90
	CD28	74.29 ± 10.85	80.61 ± 5.51^#^	76.18 ± 12.98	79.06 ± 4.49	76.72 ± 12.47	76.96 ± 6.40	72.77 ± 12.48^§^	72.28 ± 5.73^**^
CD8	CD69	3.86 ± 2.17	3.24 ± 1.07	7.72 ± 4.74^*^	4.85 ± 2.87	7.30 ± 4.56	2.91 ± 1.27^#^	6.91 ± 4.48	4.28 ± 2.01
	CD28	58.28 ± 6.97	58.40 ± 7.55	55.71 ± 6.26	55.01 ± 8.50	55.76 ± 12.36	52.21 ± 11.06	50.04 ± 11.23^*^	48.94 ± 9.94

Values represent percentages of surface marker-positive cells and are shown as mean ± SD. Women: n = 14 (13 in R+1); men: n = 8 (BDC), 9 (L-1, T5), 7 (R+1). Differences between groups were calculated using unpaired two-tailed Student’s *t* test (#). Differences between time points within the same group were calculated by one-way repeated measures ANOVA followed by *post-hoc* Holm–Sidak test (* diff to BDC; § diff to T5). *^/#/§^P < 0.05, ***/^§§§^P < 0.001.

### 3.4 Multifunctional responses of the innate and adaptive immune system

Functional capacities to mount an adequate multifunctional immune response towards stimulation were examined by immunogenic challenging with subsequent analyses of surface activation marker expression and cytokine secretion.

#### 3.4.1 Expression of surface activation markers

Incubation with the stimuli LPS and HKLM and with CD3/CD28 activation beads resulted at BDC in an upregulated expression of CD40, CD69, CD80, CD86, and TLR4 on the surfaces of monocytes. Expression of TLR2 and HLA-DR remained constant (**Table 5**, first section, CD14^+^ monocytes). The surface marker CD62L displayed intense shedding due to the incubation procedure itself, which is seen between the negative control (immediate measurement after blood draw) and basal (incubation for 6 h at 37°C). Antigen stimulation led to a further decrease of CD62L levels; however, differences to basal levels were too low to reach statistical significance (**Table 5**, second section, CD16^+^ granulocytes). CD69 was strongly induced on the surfaces of both T-cell subsets by LPS and CD3/CD28 activation beads, but CD28 expression was unaffected (**Table 5**, third section, CD4^+^ and CD8^+^ T cells). Surface activation marker expression on the distinct leukocyte subsets did not show sex-specific reaction patterns.

**Table 5:** Cell surface expression of cell activation marker on CD14^+^ monocytes, CD16^+^ granulocytes, and CD4^+^ and CD8^+^ T cells at the BDC time point after 6 h of whole-blood incubation with negative control (basal), LPS, HKLM, or CD3/CD28 activator.

Marker		Basal		LPS		HKLM		CD3/CD28	
		♀	♂	♀	♂	♀	♂	♀	♂
CD14	CD40	5.61 ± 2.67	7.57 ± 3.55	21.67 ± 9.04^***^	16.70 ± 7.32	18.31 ± 9.14	17.77 ± 8.43	13.35 ± 4.51	12.81 ± 9.86
	CD69	3.56 ± 1.84	5.46 ± 2.91	11.67 ± 6.12	13.53 ± 3.73^*^	13.22 ± 6.16^***^	11.23 ± 5.85	12.00 ± 6.32	13.00 ± 10.18
	CD80	2.57 ± 1.28	3.81 ± 2.73^***^	44.49 ± 16.53^***^	39.39 ± 16.50^*^	23.42 ± 8.10^***^	20.27 ± 8.17	15.04 ± 15.24^*^	9.99 ± 5.95
	CD86	57.42 ± 9.21	50.11 ± 9.79	82.39 ± 7.88^***^	82.31 ± 9.61^***^	83.15 ± 10.29^***^	84.53 ± 9.43^***^	84.82 ± 8.02^***^	81.44 ± 12.68^***^
	TLR2	89.45 ± 4.23	88.84 ± 8.39	83.93 ± 8.46	85.44 ± 7.94	84.60 ± 8.41	84.89 ± 9.21	87.71 ± 6.98	88.51 ± 5.19
	TLR4	9.45 ± 2.96	10.93 ± 2.62	13.83 ± 3.54^*^	15.71 ± 3.44	16.31 ± 4.81^***^	14.89 ± 6.75	15.06 ± 4.02^**^	14.73 ± 4.28
	HLA-DR	83.02 ± 5.06	81.36 ± 8.14	77.68 ± 9.63	81.02 ± 8.75	76.18 ± 9.95	77.95 ± 11.38	82.89 ± 7.13	82.92 ± 9.60
CD16	CD62L	9.01 ± 8.16	5.83 ± 5.80	4.07 ± 3.94	2.22 ± 0.69	3.32 ± 3.00^**^	2.89 ± 1.63	3.98 ± 2.18	2.62 ± 0.58
CD4	CD69	1.85 ± 1.08	2.25 ± 1.68	13.54 ± 6.92^***^	17.84 ± 7.49^***^	3.72 ± 2.28	5.08 ± 3.23	26.95 ± 18.24^***^	33.43 ± 22.60^***^
	CD28	70.11 ± 12.99	77.15 ± 7.30	64.76 ± 13.40	68.52 ± 7.97	69.64 ± 13.35	74.35 ± 6.35	n.a.	n.a.
CD8	CD69	2.95 ± 1.63	3.63 ± 1.44	23.17 ± 8.94^***^	25.47 ± 13.12^***^	6.24 ± 3.84	7.43 ± 3.98	16.09 ± 10.65^***^	27.73 ± 19.62^**^
	CD28	56.90 ± 6.45	57.90 ± 10.59	50.68 ± 10.99	50.49 ± 9.71	55.21 ± 8.32	57.35 ± 8.37	n.a.	n.a.

Values represent percentages of surface marker-positive cells and are shown as mean ± SD. Women: n = 14; men: n = 8. Differences between antigen and basal within the same group were calculated by one-way ANOVA followed by *post-hoc* Holm–Sidak test for CD14/CD86 and CD14/TLR4. For all other comparisons, the *post-hoc* Tukey test was applied. *P < 0.05, **P < 0.01, ***P < 0.001.

**Figure 4:**
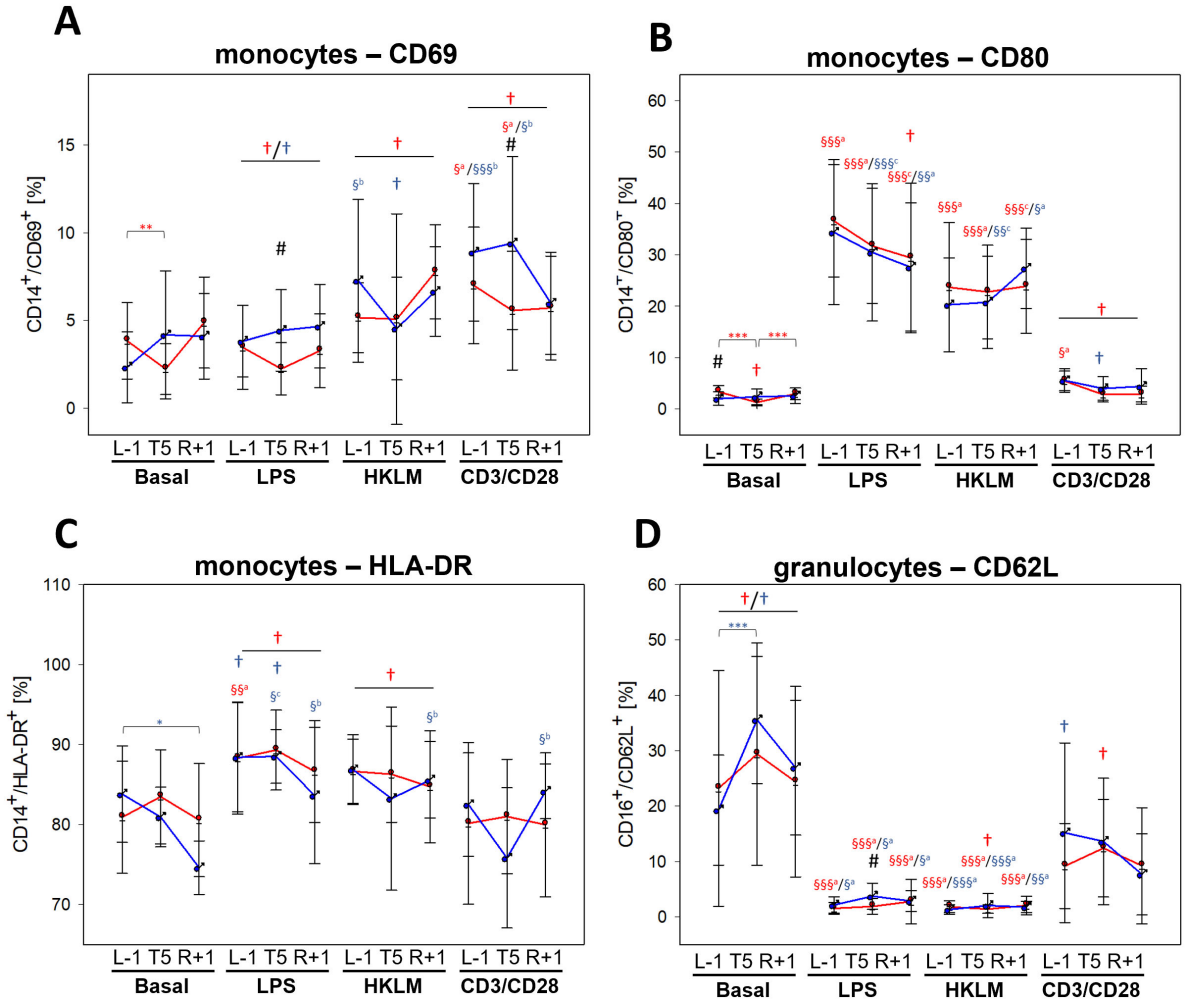
Cell surface expression of cell activation markers CD69 **(A)**, CD80 **(B)**, and HLA-DR **(C)** on CD14^+^ monocytes and CD62L on CD16^+^ granulocytes **(D)** at L-1, T5, and R+1 time points after 6-h whole-blood incubation with negative control (basal), LPS, HKLM, or CD3/CD28 activator. Values represent percentages of surface marker positive cells and are shown as mean ± SD. Females: n = 14 (13 in R+1); males: n = 9 (L-1, T5), 7 (R+1). Differences between groups were calculated using an unpaired two-tailed Student’s t test (#). Differences between antigen and basal within the same group and time point were calculated by one-way ANOVA (§) followed by *post-hoc* Tukey test (^a^), Holm–Sidak test (^b^) or Dunn’s method (^c^). Differences between time points within the same group and antigen were calculated by RM one-way ANOVA (*) as well as differences to BDC (†) followed by Holm–Sidak test. ^xxx^P < 0.05, ^xx^P < 0.01, ^x^P < 0.001.

Activation capacities of monocytes changed within the flight week encompassing L-1, T5, and R+1 time points. The increase of CD69 after incubation with all three stimuli as observed at BDC (**Table 5**) was abrogated within the flight week, irrespective of time point, with a lack of antigen-mediated increase with LPS and HKLM (**Figure 4A**). Expression of CD80 during the flight week remained unchanged to BDC after incubation with LPS and HKLM, but expression with CD3/CD28 activation beads was less pronounced than at BDC (**Figure 4B**). Conversely, surface expression levels of HLA-DR were higher after LPS and HKLM incubation during the flight week compared to BDC (**Figure 4C**). Expression levels of CD40 were significantly lower after antigen stimulation during the flight week compared to BDC, and the expression of CD86 was similar at BDC and L-1 at basal conditions. Values were significantly higher at T5 and still increased at R+1 but started to recover. Stimulation with LPS and HKLM resulted in higher CD86 surface levels during the flight week compared to BDC, which was significant for LPS. TLR2 expression was higher during the flight week than at BDC, and antigen incubation-mediated increases of TLR4 were dampened during the flight week (**Supplementary Table 2**).

In contrast to BDC, the basal incubation did not lead to lower CD62L surface levels on granulocytes during the flight week. However, activation capacities towards LPS and HKLM were comparable to BDC levels, whereas the activation state after incubation with the CD3/CD28 activator was less pronounced (**Figure 4D**).

Both CD4^+^ and CD8^+^ T cells showed no difference in basal CD69 levels between flight week and BDC. However, during the flight week, cells failed to upregulate CD69 in response to incubation with LPS, HKLM, and CD3/CD28 activator, which was similar for all three time points. CD28 expression was unaffected by antigen incubation but reduced at R+1 compared to T5 (**Supplementary Table 2**).

For surface activation marker expression on all analysed leukocytes, the factor biological sex showed no relevance.

Altogether, analysis of cell surface markers on different leukocyte subsets revealed differences between BDC and the flight week as summarized in **Table 6**; however, neither remarkable sex-specific effects were observed nor changes induced by gravitational stress.

**Table 6:** Summary of surface activation marker expression patterns on monocytes and T cells at BDC compared to the flight week.

Leukocyte subset	Surface marker	Regulation
Monocytes	CD40	↓
	CD69	↓
	CD80	**-**
	CD86	↑
	HLA-DR	↑
	TLR2	↑
	TLR4	↓
T cells	CD69	↓

#### 3.4.2 Cytokine secretion

Cytokine quantification after stimulation with LPS, HKLM, and CD3/CD28 at BDC showed stimulus-specific reactions by respective leukocyte subsets. Incubation with LPS and HKLM resulted in significant increases in G-CSF, GROα, and IL-8, all of which are involved in granulocyte development and function. Monocyte activation was reflected by increased secretion of IL-1β and IL-1RA after LPS and HKLM incubation, and higher IL-6 and TNF levels demonstrated general pro-inflammatory immune responses after stimulation. Secretion of IL-10 and IFNγ was likewise increased. Incubation with the CD3/CD28 activator significantly induced secretion of the T-cell specific cytokines IL-2 and IFNγ as well as of IL-10. Moreover, CD3/CD28 activation bead stimulation resulted in increased levels of IL-8, IL-6, IL-1RA, and TNF, but without reaching statistical significance. Cytokine secretion patterns were comparable in women and men (**Table 7**).

**Table 7:** Cytokine levels at BDC time point after 6 h of whole-blood incubation with negative control (basal), LPS, HKLM, or CD3/CD28 activator.

Cytokine	Basal		LPS		HKLM		CD3/CD28	
	♀	♂	♀	♂	♀	♂	♀	♂
G-CSF	35.86 ± 10.21	32.11 ± 10.57	556.29 ± 408.37^***^	668.22 ± 580.58^***^	200.29 ± 226.63^***^	201.22 ± 142.51^*^	38.64 ± 12.40	39.56 ± 21.30
GROα	297.79 ± 225.69	498.44 ± 970.46	3,079.07 ± 1,300.94^***^	3,760.33 ± 2,909.19^*^	2,053.00 ± 1,489.59^**^	3,149.44 ± 3,537.20^*^	787.57 ± 1,072.12	722.00 ± 893.11
IL-8	934.50 ± 774.34	1,055.56 ± 710.82	23,375.36 ± 7,114.99^***^	25,281.11 ± 6,405.20^***^	22,003.36 ± 5,619.94^***^	23,730.44 ± 7,239.18^***^	7,130.64 ± 8,491.85	6,338.00 ± 572.17
IL-6	57.07 ± 23.63	229.33 ± 473.29	40,542.79 ± 10,854.47^***^	45,374.22 ± 15,393.50^***^	24,927.86 ± 12,444.60^***^	25,079.11 ± 13,892.09^**^	1,078.29 ± 1,863.55	1,069.33 ± 2,057.87
IL-1β	42.07 ± 18.16	43.44 ± 19.73	9,809.36 ± 4,989.14^***^	11,622.78 ± 8,526.42^***^	5,714.50 ± 4,239.52^***^	6,543.33 ± 4,145.16^**^	82.43 ± 109.77	79.56 ± 87.97
IL-1RA	83.00 ± 53.25	60.11 ± 31.13	2,002.43 ± 1,055.16^***^	2,311.89 ± 1,239.05^***^	977.64 ± 967.10^***^	826.22 ± 686.53^*^	441.43 ± 469.87^*^	526.89 ± 479.34
TNF	61.21 ± 22.57	82.44 ± 53.76	13,536.43 ± 6,474.77^***^	15,451.78± 7,990.60^***^	6,488.21 ± 4,930.45^***^	9,062.11 ± 5,203.00^***^	550.93 ± 612.33	971.89 ± 1,160.10
IL-10	33.00 ± 7.18	31.11 ± 9.17	650.00 ± 443.26^***^	494.00 ± 456.84^***^	451.64 ± 575.05^***^	331.78 ± 240.62^**^	309.29 ± 244.13^**^	302.22 ± 399.67^*^
IL-2	39.64 ± 11.35	37.33 ± 11.58	49.50 ± 11.49	48.56 ± 10.39	46.14 ± 13.80	44.56 ± 13.32	185.64 ± 166.18^***^	280.22 ± 395.11^***^
IFNγ	47.93 ± 9.80	53.33 ± 13.04	2,744.71 ± 3,535.06^***^	1,846.89 ± 1,329.43^***^	1,504.14 ± 3,164.16^***^	659.78 ± 986.96^*^	654.29 ± 645.80^***^	1,450.33 ± 1,729.54^**^

Values represent MFIs (mean fluorescent intensities) and are shown as mean ± SD. Women: n = 14; men: n = 9. Differences between antigen and basal within the same group were calculated by one-way ANOVA followed by *post-hoc* Tukey test. *P < 0.05, ***P < 0.001.

**Figure 5:**
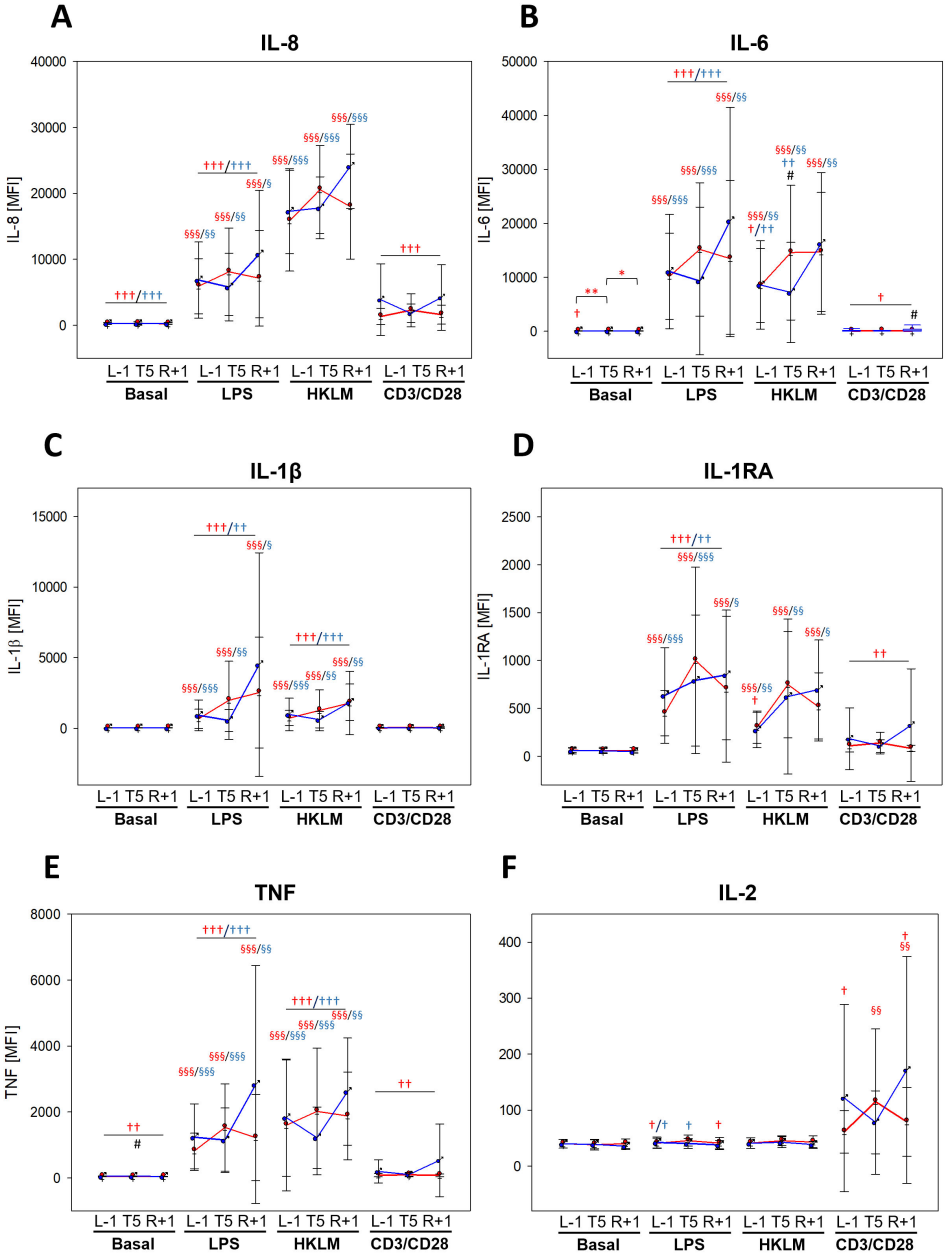
Cytokine levels at time points L-1, T5, and R+1 after 6 h of whole-blood incubation with negative control (basal), LPS, HKLM, or CD3/CD28 activator. Values represent MFIs (mean fluorescent intensities) of the cytokines IL-6, IL-8, IL-1β, IL-1RA, TNF, and IL-2 and are shown as mean ± SD. Women: n = 14 (13 at R+1); men: n = 9 (6 at R+1). Differences between groups were calculated using unpaired two-tailed Student’s t test (#). Differences between antigen and basal within the same group were calculated by one-way ANOVA followed by Tukey test (§). Differences between time points within the same group and antigen were calculated by RM one-way ANOVA (*) as well as differences to BDC (†) followed by Holm–Sidak test. ^x^P < 0.05, ^xx^P < 0.01, ^xxx^P < 0.001.

Dynamics in cytokine secretion during the flight week displayed similarities to surface activation marker analyses. While G-CSF and GROα values showed no differences between BDC and the flight week in negative control (basal) and CD3/CD28 activation beads, levels were significantly lower during the flight week in response to LPS and HKLM. However, secretion was still higher than in basal samples, which was more pronounced in women (**Supplementary Table 3**). IL-8 levels were lower in basal, LPS, and CD3/CD28 samples during the flight week than at BDC but remained unaffected with HKLM (**Figure 5A**). Levels of IL-6, IL-1β, and IL-1RA (**Figures 5B–D**) were altogether comparable between BDC and flight week; however, secretion in response to antigen stimulation was lower (IL-6, IL-1β, and IL-1RA for LPS; IL-6 and IL-1β for HKLM; IL-6 and IL-1RA for CD3/CD28). TNF secretion was likewise strongly affected, showing a reduction already in negative controls and towards all antigens during the flight week compared to BDC (**Figure 5E**). Cytokines with a high T-cell specificity showed no measurable differences in basal and HKLM samples between BDC and the flight week, but these cytokine responses were significantly dampened in response to LPS and CD3/CD28 (**Figure 5F**, **Supplementary Table 3**).

Although a majority of the measured cytokines displayed lower antigen-mediated secretion in the flight week than at BDC, reactivity towards the applied stimuli was predominantly maintained. Nevertheless, sex-specific differences or changes induced by PF were not identified.

### 3.5 Influence of stress hormones

**Figure 6:**
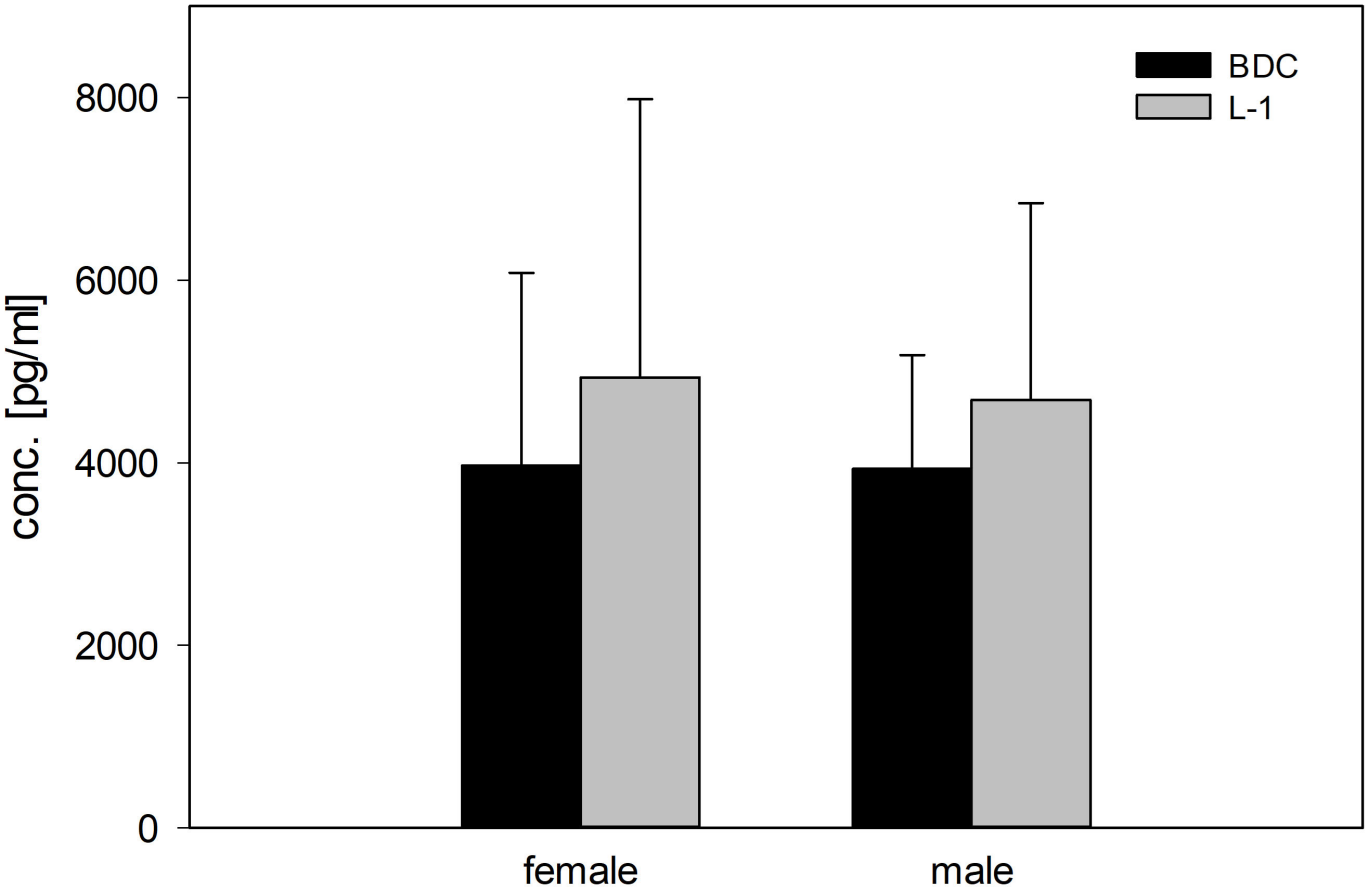
Concentrations (pg/ml) of the stress hormone cortisol (morning values) in saliva in female and male study participants at BDC (black bars) and L-1 time points (grey bars).

Salivary concentrations of the stress hormone cortisol showed no significant differences between the BDC and L-1 time points, although concentrations tended to be higher at L-1 in both groups (**Figure 6**).

## 4 Discussion

Being aware that biological sex represents an important variable in immunity and in view of increasing numbers of female astronauts and the facilitated access to spaceflight missions by commercial providers, the main aim of this study was to elaborate on functional changes of innate and adaptive immune cells after acute gravitational stress induced by parabolic flight (PF) with a special focus on sex-specific differences.

The present study delivered two meaningful findings that will be addressed separately in the following sections. Firstly, exposure to acute gravitational stress by PF does not have a sustaining impact on the immune functions investigated herein and the responses were similar regarding direction and intensity of reaction in women and men. Secondly, leukocyte activation capacities towards immunogenic challenging differed between the flight week and BDC, which took place 1 month before the actual event.

### 4.1 Lack of impact of the factor acute gravitational stress and the variable biological sex

It is commonly known that women and men differ in innate and adaptive immune responses, both with regard to immune cell abundances and activity which is heavily attributed to sex hormones and X-linked immune-related genes (24, 25). Men for instance show higher CD8^+^ T-cell and NK cell numbers. Women on the other side display elevated CD4^+^ T-cell and B-cell levels and more efficient functional properties such as phagocytosis and antigen-presentation, which altogether results in increased pathogen clearance and vaccine efficacy in women (19, 20, 22).

In detail, the results of the presented experiments show altered leukocyte subset distributions after PF with elevated proportions of neutrophilic granulocytes and concomitant reductions in monocyte and lymphocyte proportions, leading to elevated NLRs at T5. Changes in NLRs reflect alterations in overall immune state, with elevated levels being considered as indicators for inflammation and neutrophil activation (26, 27).

Interestingly and directly related to spaceflight conditions, elevated NLRs were also demonstrated at the late phase of prolonged space flight as well during hindlimb unloading (HU), a microgravity-simulating approach in rodents. In both settings, elevated NLRs were suggested to be associated with increasing oxidative stress during mission or HU, respectively (26). However, elevated NLRs were also observed immediately after return to Earth and after long-term bedrest.

Increased granulocyte proportions in general are in accordance with results from other studies in PF, which are altogether attributed to an increased release of neutrophils into the blood stream caused by the experienced acute physical or gravitational stress (17, 27, 28). Acute stress is known to temporarily boost immune defenses, especially by mobilizing neutrophils from bone marrow and peripheral tissues with high capacities for pathogen killing by oxidative burst and phagocytosis (29). A higher readiness to perform oxidative burst was demonstrated during and after PF. In an *in vitro* approach using rat alveolar macrophages Adrian and colleagues showed that release of reactive oxygen species (ROS) by oxidative burst is highly dependent on gravity levels, being reduced in microgravity and reinforced in hypergravity (14). Another study focused on PMNs (polymorphonuclear granulocytes) that were freshly isolated from human blood after study subjects were exposed to PF. This *ex vivo* approach demonstrated a primed state of PMNs with increased H_2_O_2_ production in response to soluble stimuli such as PMA, which lasted for at least 48 h (17). However, neither the present investigations nor the study performed by Kaufmann et al. detected a PF-mediated effect on phagocytosis (30). Results obtained by investigations on NETosis activity in the present experimental setting suggests that activating properties of gravitational stress only apply to distinct cellular mechanisms. Altogether, this supports the conclusion that oxidative burst is a highly gravity-sensitive process (14), whereas other innate immune functions can be likewise impacted by stress, but not by acute changes in gravity levels.

The assessment of PF on general leukocyte activation states was conducted by quantification of surface activation marker expression before (BDC, L-1) and after PF (T5, R+1) on monocytes, granulocytes, and T cells. Changes occurred only sporadically without showing a clear and comprehensible pattern of increased or decreased cell activation, indicating a negligible effect of acute gravitational stress on this parameter. Of note, the T5 time point has to be additionally evaluated under the light of possible effects of scopolamine, which has been shown in rodent studies to promote neuroinflammation and to provoke Alzheimer’s disease (31–33) but to also affect inflammatory processes outside the central nervous system (34). However, within the investigation presented herein, no relevant alteration of unstimulated plasma cytokines occurred between pre- and post-flight, indicating a negligible impact of scopolamine in this case. Nevertheless, one cannot definitively exclude any scopolamine effect in humans on inflammation and thus, future studies need to determine the scopolamine effect in the setting of a parabolic flight.

To obtain insights into affected capabilities of mounting an adequate multifunctional immune response towards immunogenic challenging after gravitational stress, whole-blood incubation assays were performed using the Gram-negative bacterial component LPS, HKLM as representative for Gram-positive bacteria, and CD3/CD28 activation beads, specifically acting on the T-cell receptor. In the course of all three flight week time points (L-1, T5, and R+1), surface marker expression levels as well as secreted cytokine concentrations in response to antigen stimulation were found comparable, suggesting that gravitational stress has no priming effect towards subsequent immunogenic challenging. This is corroborated by results that derive from previous investigations encompassing *ex vivo* whole blood incubation assays within a PF campaign, in which a simultaneous treatment elicited intensified reactions of immune cells; a subsequent antigen incubation after PF, however, failed to induce immune responses that deviated from ground control samples (18). These observations suggest that acute gravitational stress causes immediate adaptations of immune cells (35) which are quickly reversible after re-entering 1*g* normogravity. In relation to this, it should be kept in mind that the duration between the last flown parabola and blood draw, as well as the start of experiments, was approximately 1.5 h which would provide enough time to re-adapt to normogravity conditions.

Furthermore, the immune reactions investigated herein were similar in women and men regarding trend and intensity, demonstrating that despite the well-known particular differences in immunity, there are no sex-specific higher risks of suffering from dysregulated immune responses after short-term gravitational stress. A similar course has also been observed in a chronic stress model during isolation studies in Antarctica (21). Both outcomes are in discordance with the general understanding of stronger immune responses in women. Presumably, the short experiment durations of 6 and 48 h (21) were too short to elicit sex-specific differences. To further consolidate these observations, higher group sizes should be considered in future investigations in order to increase statistical power.

### 4.2 Pronounced differences in cell activation between BDC and flight week

Activation marker expression on monocytes displayed an expression pattern that indicates two controversial activation states. On the one hand, a reduced activation is shown by decreased CD40 (36), CD69 (37), and TLR4 levels (38). On the other hand, prerequisites for monocyte-mediated T-cell activation were enhanced as shown by persistent or higher expression levels of the co-stimulatory molecules CD80 and CD86 (39, 40) as well as of HLA-DR levels, which are crucial for antigen presentation (41). These changes, however, did not result in elevated antigen-mediated T-cell activation as mirrored by CD69 expression levels, which were lower during the flight week than at BDC. At this point, the question remains if these contrary activation states of monocytes may serve as a kind of compensatory mechanism in response to other downregulated immune functions. Interestingly, the pronounced granulocyte activation as shown by intense CD62L shedding (30) at BDC, which occurred already in negative control (basal) samples in whole-blood incubation assays, and therefore represented a high susceptibility to mechanical stress, was remarkably reduced within the flight week, suggesting an overall reduction in granulocyte activation readiness. The supposed attenuated intensity in immune responsiveness was further corroborated by secreted cytokine patterns. Although concentrations were specifically increased through antigen incubation compared to basal values, concentrations during the flight week were clearly lower than at BDC, regardless of cytokine specificity.

The reasons for these alterations are not clear and remain a point of discussion. One possible explanatory approach might be represented by release of the stress hormones having dampening actions on immune responses (11, 42, 43). However, measured cortisol levels were comparable at BDC and onset of flight week, making this stress hormone unlikely as a potential repressive candidate. In addition, a lack of increase in cortisol levels after PF was also documented by others (28). Unfortunately, assessing the levels of the stress mediator norepinephrine, which is released due to excitement (44) and has a proven downregulating impact on distinct innate immune cell functions (43), was not included in the present investigation. However, results from previous *ex vivo* investigations from our group indicate that norepinephrine levels have no effect on the immune response in a short-duration whole-blood incubation approach (21, 43).

Another explanation for weaker immune cell responsiveness might be the effect of travelling out of everyday life, which was moreover dominated by the COVID-19 pandemic in this study. Expectancy of the flight week and associated positive anticipatory stress regulation as well as chronic mild stress could have led to persistent slightly elevated cortisol levels between BDC and L-1, which can prime the body to the acute PF stress event (45, 46). As a consequence, cortisol concentrations, which were slightly higher at L-1 than at BDC, experienced only a marginal increase upon exposure to the actual acute stress event of PF. In this case, immune functions could have been sustainably affected and thereby weakened (47, 48). Future investigations should therefore include repetitive pre-flight week time points to assess a possible effect of anticipatory or mild chronic stress, respectively. Sleep behavior might have been also affected, which in turn has a direct impact on immune functions (49, 50). To our knowledge, dynamics on the cell functional level at the beginning of a journey and a potentially enhanced susceptibility towards infections were not examined by now and thus represent an important subject of future investigations.

### 4.3 Conclusion

Acute gravitational stress induced by PF affects only distinct immune functions sustainably, such as oxidative burst. Moreover, immune cells display a high potential to quickly adapt to prevailing *g*-levels. The tests performed in this study did not provide evidence for a different impact of acute gravitational stress on basic innate and adaptive immune functions in women and men. However, to assess sex-specific differences with relevance for long-term spaceflight missions, descriptive and functional analyses with the focus on biological sex during present missions in LEO are needed.

### 4.4 Limitations

We acknowledge the limitations that performing functional tests on immune cell capacities was only possible on ground and thus an immediate effect of gravitational stress could not be assessed. Due to this fact and the specific flight circumstances, circadian alterations in immune response could not be investigated as well. The field of circadian immunity is rapidly increasing, and it was nicely shown that the immune system is very time-of-day dependent (51). However, for the present study, one has to assume that the forces of a parabolic flight have a higher impact than the daily circadian alterations on immunity, as already observed for cortisol levels, for which the circadian rhythm of peaking in the morning and a gradual decline over time of the day was neutralized in parabolic flight (52).

Due to technical constraints and cancellation of the first flight day and a catch-up on the following day, the full set of analysis parameters and time points could not be fulfilled for all study subjects, leading to different subject numbers between the time points.

## Data Availability

The raw data supporting the conclusions of this article will be made available by the authors, without undue reservation. Supplementary information accompanies this manuscript and is attached as a single file. The original data can be made available upon reasonable request.
