# Supplementary material for: Biological sex as a variable in immunity does not affect parabolic flight-induced alterations in immune responses
Source: Front Immunol. 2025 Nov 17;16:1673072. doi: 10.3389/fimmu.2025.1673072 (PMC12665576; doi:10.3389/fimmu.2025.1673072)
Supplement: Supplementary file 1 [file DataSheet1.pdf]

Biological Sex as a Variable in Immunity does not affect Parabolic Flight-induced alterations in immune responses

Dominique Moser[1], Judith-Irina Buchheim[1], Katharina Biere[1], Sandra Matzel[1], Federico D´Amico[1], Alexander Choukér[1], Tobias Woehrle[1], Matthias Feuerecker[1]

[1]Laboratory of Translational Research 'Stress and Immunity', Department of Anesthesiology, LMU Hospital, Ludwig-Maximilians-University Munich, Munich, Germany

Supplementary information consists of

Supplementary Figure 1

Supplementary Table 1

Supplementary Table 2

Supplementary Table 3

Supplementary Figure 2

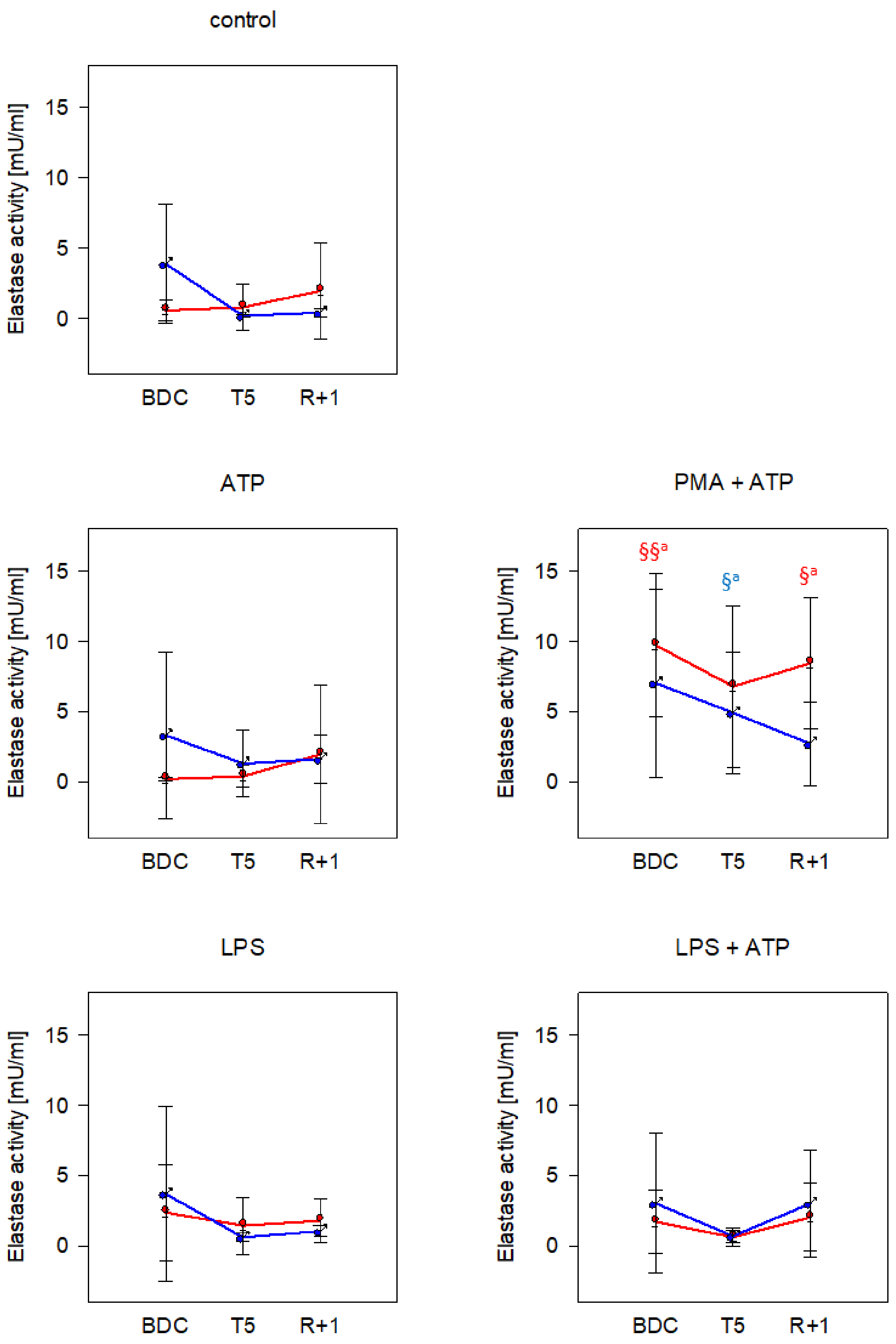

Supp. Figure 1 NETosis intensity at time points BDC, T5 and R+1 quantified by neutrophil elastase activity [mU/ml]. Neutrophils were incubated for three hours with ATP, PMA+ATP, LPS and LPS+ATP for NETosis induction. Values show mean ± SD for women (red lines, n=9) and men (blue lines, n=5). Differences between negative control and stimuli within the same group were calculated by one-way ANOVA followed by post hoc Tukey test ([a]) or Holm-Sidak ([b]) test. $^{§}P < 0.05$, $^{§§}P < 0.01$.

Supp. Table 1 Proportions of B cells among all lymphocytes in whole blood at the respective sample collection time points using IMK multitest. Values are given as mean ± SD (red, n=14 (BDC, L-1, T5), n=13 (R+1)) and men (blue, n=9 (BDC, L-1, T5), n=6 (R+1)). Differences between groups were calculated using unpaired two-tailed Student´s *t* test (#). Differences to T5 within the same group were calculated by one-way repeated measures ANOVA (*) followed by post hoc Holm-Sidak test. *P < .05, $^{\#\#}P$ < .01.

	**BDC**		**L-1**		**T5**		**R+1**	
	♀	♂	♀	♂	♀	♂	♀	♂
CD19	7.70 ± 2.33*	11.65 ± 2.87$^{\#\#}$	7.84 ± 3.09*	9.91 ± 2.57	9.75 ± 3.58	12.53 ± 3.31	8.34 ± 3.39	11.52 ± 3.59

Supp. Table 2 Cell surface expression of cell activation markers on CD14^{+} monocytes, CD16^{+} granulocytes, and CD4^{+} and CD8^{+} T cells at time points L-1, T5 and R+1 after 6 hours whole blood incubation with negative control (basal), LPS, HKLM or CD3/CD28 activator. Values represent percentages of surface marker positive cells of and are shown as mean ± SD. Women: n = 14; men: n = 8 (6 in R+1). Differences between groups were calculated using unpaired two-tailed Student´s t test (#). Differences between antigen and basal within the same group were calculated by one-way ANOVA (§) followed by post hoc Tukey test (^{a}), Holm-Sidak test (^{b}) or Dunn´s method (^{c}). Differences of L-1 and R+1 to T5 (*) as well as differences to BDC (†) within the same group and antigen were calculated by RM one-way ANOVA followed by post hoc Holm Sidak test. ^{x}P < 0.05, ^{xx}P < 0.01, ^{xxx}P < 0.001.

			**L-1**		**T5**		**R+1**	
			♀	♂	♀	♂	♀	♂
**Basal**	CD14/	CD40	8.34 ± 3.26*	6.39 ± 2.98	4.93 ± 2.52	8.50 ± 6.45	9.89 ± 3.33***,†	8.32 ± 5.41
		CD86	55.38 ± 14.56***	48.38 ± 9.58	72.99 ± 10.71†††	65.04 ± 18.63	67.03 ± 11.54†	61.38 ± 9.05
		TLR2	90.23 ± 4.13*	93.46 ± 2.80	94.36 ± 3.23††	93.72 ± 2.64	91.52 ± 2.72	91.30 ± 5.66
		TLR4	8.99 ± 2.70	6.91 ± 2.02	9.22 ± 3.71	11.70 ± 6.08	9.34 ± 3.28	8.19 ± 2.04
	CD4	CD69	1.72 ± 0.98	1.40 ± 0.92	2.04 ± 1.54	2.93 ± 1.97	2.32 ± 1.48	1.67 ± 0.53
		CD28	70.26 ± 14.98	74.99 ± 4.07	72.06 ± 11.50	76.25 ± 3.84	65.27 ± 12.30*	69.06 ± 5.39**,†††
	CD8	CD69	5.22 ± 4.08	3.18 ± 1.57	4.77 ± 3.66	4.89 ± 4.47	4.95 ± 2.56	4.29 ± 1.89
		CD28	59.58 ± 9.35	61.09 ± 9.18	61.37 ± 8.29	62.14 ± 8.40	54.678 ± 10.85*	57.95 ± 10.36
**LPS**	CD14/	CD40	6.39 ± 4.41†††	7.02 ± 3.67†††	3.88 ± 2.32†††	8.75 ± 5.63$^{†††,\#}$	6.02 ± 3.25†††	9.86 ± 5.55†††
		CD86	91.09 ± 2.95^{†††,§§§,a}	90.70 ± 7.06^{†,§§§a}	91.56 ± 3.80^{†††,§§§,a}	92.81 ± 4.10^{†,§§§,c}	90.71 ± 4.62^{†††,§§§,a}	87.14 ± 7.26^{§§§,b}
		TLR2	94.22 ± 2.71^{†††,§,a}	93.19 ± 5.70†	96.50 ± 2.28†††	95.62 ± 2.36††	95.06 ± 2.53^{†††,§,b}	92.53 ± 5.30

		TLR4	9.00 ± 2.13[†††]	9.74 ± 4.22[††]	8.09 ± 1.85[†††]	9.74 ± 2.70[††]	9.62 ± 4.12[††]	11.63 ± 4.38
	CD4	CD69	3.81 ± 7.05[†††]	4.19 ± 3.22[†††]	3.28 ± 2.33[†††]	2.75 ± 0.99[†††]	3.08 ± 2.68[†††]	7.29 ± 8.25[†††]
		CD28	68.83 ± 14.60	73.11 ± 3.67	70.25 ± 11.80	73.24 ± 6.76	64.20 ± 12.07[*]	67.34 ± 10.25
	CD8	CD69	12.10 ± 12.10[†††]	8.27 ± 5.80[†††]	7.72 ± 5.89[†††]	7.07 ± 4.21[†††]	9.09 ± 6.95[†††]	15.41 ± 13.66[†]
		CD28	57.39 ± 9.93[†]	57.80 ± 9.06	60.31 ± 9.51[††]	59.95 ± 10.44[†]	54.62 ± 11.43	53.42 ± 10.39
**HKLM**	CD14/	CD40	7.38 ± 3.32[†††]	8.23 ± 3.31[†]	5.63 ± 4.64[†††]	9.31 ± 6.91	12.04 ± 7.31[*]	11.44 ± 5.78
		CD86	89.27 ± 3.35[§§§a]	87.94 ± 4.45[§§a]	89.18 ± 4.94[§§§,a]	88.99 ± 2.53[§,c]	88.70 ± 4.05[§§§,a]	87.64 ± 5.36[§§§,b]
		TLR2	93.49 ± 2.95[†††]	94.12 ± 3.74[††]	96.42 ± 2.06[†††]	94.97 ± 3.46[††]	92.58 ± 3.70[††]	92.46 ± 6.06[†]
		TLR4	9.27 ± 2.49[†††]	10.93 ± 3.35[§,a]	11.63 ± 4.49[††]	15.43 ± 9.35	10.79 ± 2.58[††]	9.87 ± 2.36
	CD4	CD69	1.10 ± 0.44[†††]	1.56 ± 0.94[††]	1.65 ± 1.49[†††]	2.46 ± 1.74[†]	2.46 ± 1.41	3.16 ± 1.38
		CD28	68.85 ± 14.73	72.62 ± 4.58	71.56 ± 11.45	74.91 ± 6.31	64.16 ± 12.81[***,†]	68.90 ± 8.21
	CD8	CD69	3.92 ± 3.48	3.04 ± 2.88[††]	3.12 ± 2.17[†]	3.84 ± 3.22[†]	3.99 ± 3.38	6.86 ± 4.70
		CD28	59.04 ± 9.42	59.09 ± 9.06	60.69 ± 11.12	62.35 ± 11.31	53.99 ± 10.86	51.47 ± 11.44[*]
**CD3/CD28**	CD14/	CD40	10.70 ± 4.17	11.31 ± 2.17[§§,b]	7.71 ± 2.83[††]	10.48 ± 3.72	9.58 ± 4.69[†]	8.42 ± 5.60
		CD86	79.72 ± 11.91	72.34 ± 18.28	83.40 ± 7.37	76.87 ± 8.55	80.73 ± 11.30	79.70 ± 6.77[§§,b]
		TLR2	90.04 ± 5.32	91.78 ± 3.85	94.18 ± 4.24[††]	92.97 ± 2.95[†]	94.08 ± 3.69[††]	92.85 ± 4.50[†]
		TLR4	12.25 ± 4.39	11.54 ± 2.87[§§,a]	10.24 ± 3.13[††]	13.36 ± 5.30	10.07 ± 3.23[††]	11.19 ± 3.31
	CD4	CD69	6.60 ± 7.09[†††,§§,a]	7.74 ± 3.77[†††,§§§,a]	8.03 ± 7.70[†††,§§,a]	7.41 ± 4.61[†††,§,a]	7.55 ± 6.31[†††,§§,a]	12.26 ± 9.54[††,§,a]
		CD28	n.a.	n.a.	n.a.	n.a.	n.a.	n.a.
	CD8	CD69	7.59 ± 7.01[††]	5.72 ± 2.46[†††]	7.92 ± 5.58[††]	6.83 ± 3.42[††]	8.14 ± 6.09[††]	8.11 ± 4.84[††]
		CD28	n.a.	n.a.	n.a.	n.a.	n.a.	n.a.

Supp. Table 3 Cytokine levels at timepoints L-1, T5 and R+1 after 6 hours whole blood incubation with negative control (basal), LPS, HKLM or CD3/CD28 activator. Values represent MFIs (mean fluorescent intensities) of the cytokines G-CSF, GROα, IL-10, and IFNγ and are shown as mean ± SD. Women: n = 14 (13 at R+1); men: n = 9 (6 at R+1). Differences between groups were calculated using unpaired two-tailed Student´s t test (#). Differences between antigen and basal within the same group were calculated by one-way ANOVA (§) followed by post hoc Tukey test (a) or Holm-Sidak test (b). Differences between flight week time points and BDC within the same group and antigen were calculated by RM one-way ANOVA followed by post hoc Holm Sidak test (†).$^{x}P < 0.05$, $^{xx}P < 0.01$, $^{xxx}P < 0.001$.

		L-1		T5		R+1	
		♀	♂	♀	♂	♀	♂
**Basal**	G-CSF	34.71 ± 7.48	35.33 ± 12.45	35.21 ± 9.53	35.11 ± 8.45	35.54 ± 8.33	33.33 ± 10.33
	GROα	317.50 ± 190.33	488.33 ± 764.83	286.14 ± 286.33	439.00 ± 597.81	278.69 ± 118.77	465.83 ± 732.29
	IL-10	33.07 ± 9.09	44.22 ± 26.77	31.86 ± 8.40	32.89 ± 6.94	34.69 ± 4.99	36.50 ± 6.92
	IFNγ	48.43 ± 7.84	81.56 ± 58.68$^{\#}$	45.00 ± 8.48	67.78 ± 52.61	45.23 ± 7.42	48.67 ± 4.13
**LPS**	G-CSF	44.86 ± 12.20†††	59.00 ± 45.12††	74.36 ± 66.52†††	63.67 ± 82.85††	66.31 ± 41.20†††	186.67 ± 289.92†
	GROα	464.36 ± 223.58†††	680.44 ± 1015.22††	664.79 ± 455.92^{†††,§§,a}	509.67 ± 455.20†††	470.54 ± 205.75†††	1207.67 ± 1504.25††
	IL-10	56.14 ± 22.42^{†††,§,a}	64.56 ± 44.34†	139.29 ± 180.14^{†††,§,a}	87.78 ± 154.11†	171.08 ± 202.73†††	118.17 ± 94.11†
	IFNγ	57.57 ± 16.57††	96.44 ± 61.85†††	65.29 ± 27.40††	101.78 ± 98.89†††	70.15 ± 40.51††	174.83 ± 160.40†††
**HKLM**	G-CSF	49.29 ± 17.09^{††,§,b}	58.67 ± 47.24††	94.93 ± 99.15^{§§,b}	79.67 ± 82.11†	74.15 ± 34.58^{†,§,a}	100.83 ± 57.13
	GROα	639.71 ± 414.94^{†††,§,a}	1091.67 ± 1738.97	1046.43 ± 753.87^{†,§§§,a}	775.00 ± 552.99†	958.46 ± 583.75^{††,§§,a}	729.50 ± 355.62†
	IL-10	70.86 ± 38.24^{§§,a}	66.33 ± 39.96	245.07 ± 372.36^{§§§,a}	168.89 ± 304.42	200.46 ± 181.40^{§§§,a}	246.83 ± 208.63
	IFNγ	64.57 ± 19.85	97.44 ± 59.08	78.00 ± 31.04^{§§§,a}	99.11 ± 65.44^{§,a}	71.46 ± 18.21^{§§,a}	102.00 ± 33.89$^{\#}$
**CD3/CD28**	G-CSF	35.43 ± 8.21	34.44 ± 8.59	37.71 ± 9.67	34.44 ± 9.94	35.85 ± 6.90	34.00 ± 8.00
	GROα	370.00 ± 177.91	542.11 ± 885.90	352.14 ± 269.89	421.78 ± 480.20	312.85 ± 211.02	440.67 ± 650.38
	IL-10	54.29 ± 32.80†††	91.11 ± 95.28	106.57 ± 122.27†††	63.44 ± 52.56	74.92 ± 62.14†††	135.83 ± 150.30
	IFNγ	132.71 ± 114.38^{†††,§§§,a}	393.22 ± 734.29^{§,a}	147.14 ± 129.76^{†††,§§§,a}	188.78 ± 157.94^{§,a}	102.46 ± 52.71^{†††,§§§,a}	1153.17 ± 2410.75

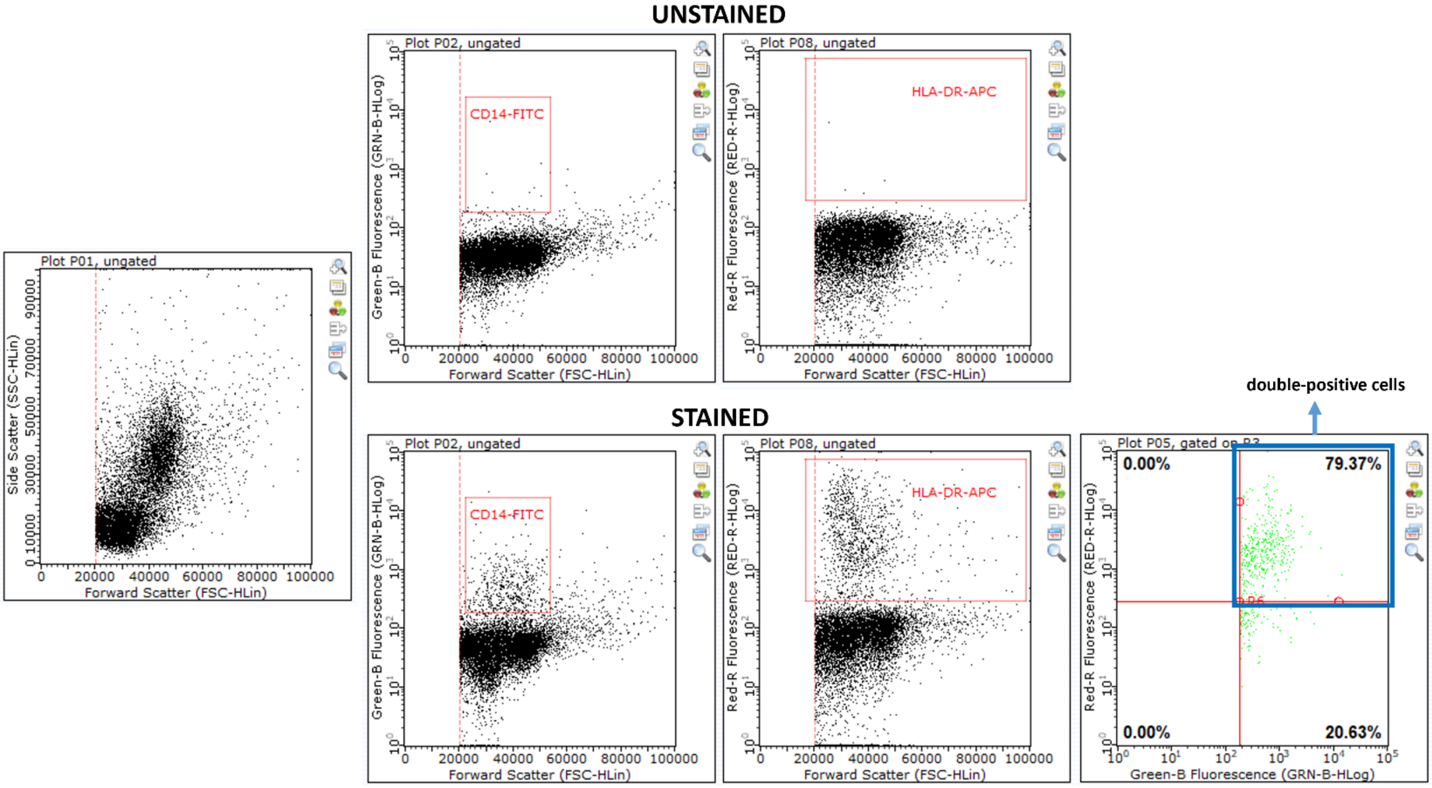

Supp. Figure 2 Exemplary gating strategy for immunophenotyping (CD14-FITC/HLA-DR-APC double-positive cells).
